# Development and evaluation of a risk prediction model for social disability in schizophrenia patients

**DOI:** 10.3389/fpsyt.2025.1588849

**Published:** 2025-10-07

**Authors:** Xiaojian Jiang, Tingting Xiang, Sally Wai Chi Chan, Yang Han

**Affiliations:** ^1^ School of Nursing, Hunan University of Chinese Medicine, Changsha, Hunan, China; ^2^ Hunan Provincial Maternal and Child Health Care Hospital, Changsha, Hunan, China; ^3^ Tung Wah College, Hong Kong, Hong Kong SAR, China; ^4^ School of Civil Engineering, Central South University, Changsha, Hunan, China

**Keywords:** schizophrenia, social disability, risk prediction model, medication adherence, cognitive function

## Abstract

**Background:**

Schizophrenia is a severe mental disorder with a significant impact on social functioning. Social disability is common in patients, requiring a reliable prediction model for early intervention. This study aimed to develop and validate a risk prediction model for social disability in schizophrenia patients, focusing on key contributing factors.

**Methods:**

A cross-sectional study that involved 473 schizophrenia patients was conducted between February and September 2021. Standardized assessments, including the Social Disability Screening Schedule, Brief Psychiatric Rating Scale, The Medication Adherence Report Scale, and Brief Assessment of Cognition in Schizophrenia, were administered. Logistic regression was employed to identify the independent risk factors for social disability, and the model performance was evaluated using the area under the receiver operating characteristic curve (AUC) and the Hosmer–Lemeshow goodness-of-fit test.

**Results:**

Among the 473 participants (56.0% male, mean age = 29.31 ± 8.7 years old), 314 (66.4%) had a social disability. Significant differences in educational level, income, residence, and clinical characteristics were observed between the social disability and non-disability groups. The multivariate logistic regression analysis identified six independent risk factors for social disability: severity of psychiatric symptoms, medication adherence, cognitive function, perceived stigma, social support, and psychological capital. The final risk prediction model demonstrated strong discriminatory ability, with an AUC of 0.860 (95% CI: 0.820–0.899). The model exhibited high sensitivity (0.873) and specificity (0.868), with good calibration, as indicated by the Hosmer–Lemeshow test (*X^2^* = 5.746, *p* = 0.783).

**Conclusions:**

The risk prediction model can effectively identify schizophrenia patients at high risk for social disability, supporting early and targeted interventions to improve outcomes.

## 1 Introduction

Schizophrenia is a severe and complex mental disorder, and its exact causes remain unclear. Globally, the prevalence of schizophrenia ranges between 0.33% and 0.75% (1). In China, over 8 million adults were diagnosed with the condition, contributing to a prevalence rate of 1.12% (2, 3). These figures highlight the significant societal and health burden posed by schizophrenia, underscoring the need for targeted research and intervention strategies to better manage the disorder.

One of the significant challenges faced by patients with schizophrenia is social disability, which is characterized by impaired social functioning, and the inability to fulfill social responsibilities (4, 5). The biopsychosocial medical model, which is widely accepted in modern mental health care, emphasizes the importance of reintegrating patients into society as part of their recovery process (6). Restoring social functioning is crucial for achieving this goal. However, a number of patients, especially long-term inpatients, continue to struggle with significant social function impairments (7, 8).

The development of social disability in schizophrenia patients is influenced by a complex interplay of factors (9). Long-term hospitalization and the chronic nature of the illness contribute to the high risk of social dysfunction. As the importance of prevention gains more attention, tailored psychological and social interventions have been proven to be effective in improving or delaying the progression of social disability in patients with schizophrenia. Thus, the development of a risk prediction model is essential, to better address this issue. Such a model would help to identify patients who are most at risk of developing social disability, allowing for early and personalized interventions. These efforts are crucial in reducing the prevalence of social disability, and improving the overall quality of life for patients with schizophrenia.

The present study aims to develop a risk prediction model for social disability in schizophrenia patients, focusing on the key factors that contribute to social disability. This model would facilitate its early detection and targeted interventions, ultimately enhancing patient outcomes in the long term.

## 2 Materials and methods

### 2.1 Study design and participants

The present study employed a cross-sectional approach to identify the risk factors associated with social disability in schizophrenia patients. Patients were recruited using a convenience sampling approach from one tertiary hospital, which was selected from among three accredited institutions providing schizophrenia treatment in Changsha, Hunan Province, China. Both outpatients and inpatients who met the inclusion criteria were consecutively enrolled between February and September 2021.

Participants were eligible for inclusion if they met the following criteria: (1) aged 18 years or older; (2) diagnosed with schizophrenia in accordance with either the Chinese Classification of Mental Disorders, 3rd Edition (CCMD-3), or the International Classification of Diseases, 11th Revision (ICD-11), with diagnostic confirmation based on consensus between two certified psychiatrists; (3)Patients were considered mentally stable if they were deemed so by the attending psychiatrist and had a total PANSS score of less than 80 within one week before participation (10, 11); and (4) provided informed consent and agreed to participate in the study. Exclusion criteria included: (1) the presence of acute or severe psychotic symptoms; (2) a history of organic brain injury or neurological disorders; (3) substance-induced mental disorders such as alcohol or drug dependence; and (4) intellectual disabilities.

Through a comprehensive review of the literature, 25 potential risk factors related to social disability in schizophrenia were identified (4, 6, 7). Based on logistic regression analysis principles, where each independent variable requires a sample size of 5–10 patients (12), and given a reported prevalence of social disability in schizophrenia patients in China of approximately 70–80% (9), the study aimed to recruit at least 357 participants. This number was adjusted to account for a 10% likelihood of invalid questionnaires, resulting in a final estimated sample size of 397 participants.

### 2.2 Procedure

#### 2.2.1 Preparation before data collection

Prior to the formal commencement of data collection, a multidisciplinary research team was assembled, comprising one doctoral supervisor specializing in mental health research, two chief psychiatrists, one psychiatric nurse, and three master’s students in nursing with a focus on mental health. The roles were clearly delineated: the doctoral supervisor oversaw the study design and implementation; the chief psychiatrists were responsible for evaluating participants’ psychiatric symptoms; the psychiatric nurse and the master’s students facilitated questionnaire administration and data collection. Before initiating data collection, all team members participated in a structured one-week training program designed to ensure methodological consistency and data quality. The training covered the following topics: (1) the concept and clinical relevance of social functional impairment in schizophrenia; (2) symptom identification and assessment procedures; (3) standardized scoring of the study questionnaires; (4) ethical and methodological considerations in survey administration; and (5) procedural precautions to ensure participant safety and data integrity. At the conclusion of the training, team members completed a brief knowledge assessment to confirm understanding and readiness for field implementation. Only those who passed the evaluation were authorized to participate in the data collection process.

#### 2.2.2 Data collection process

Before administering the questionnaires, investigators held face-to-face discussions with each participant and their family members to explain the purpose and scope of the study, clarify the content of each instrument, and ensure participant comprehension. Informed consent was obtained in writing from all participants. During the administration of the questionnaires, investigators adhered strictly to standardized instructions and refrained from offering any subjective interpretations or guidance, in order to maintain procedural consistency and reduce interviewer bias.

The Social Disability Screening Schedule (SDSS), Insight and Treatment Attitude Questionnaire (ITAQ), Social Support Rating Scale (SSRS), Perceived Devaluation-Discrimination Scale (PDD), Positive Psycap Questionnaire (PPQ), and the Positive and Negative Affect Scale (PANAS) were administered using an assisted self-report approach. For participants with low literacy levels (i.e., fewer than six years of formal education), standardized assistance was provided by trained interviewers. This assistance involved reading each questionnaire item aloud in a neutral tone, carefully avoiding any leading or suggestive interpretations. After each item was presented, participants were asked to verbally confirm their selected responses to ensure accurate understanding and reliable data collection. The Medication Adherence Report Scale (MARS-5) and the Brief Assessment of Cognition in Schizophrenia (BACS) were completed by the investigators based on the available medical information.

#### 2.2.3 Quality control

To minimize response bias, particularly in sensitive areas, the following measures were implemented: (1) Prior to completing the questionnaires, interviewers conducted detailed discussions with participants to emphasize the confidentiality of their responses; (2) Participants were explicitly informed that their personal information would be protected and not disclosed to anyone outside the research team; (3) All questionnaires were anonymized, and participants were reassured that there were no “correct” or “incorrect” answers—only honest responses based on personal experiences; (4) A clear statement on the cover page of the questionnaire indicated that the survey was intended solely for clinical research and that all personal information would remain confidential; and (5) Completed questionnaires were collected by trained interviewers, assigned unique identification codes, and stored securely. These procedures were designed to enhance methodological rigor and reduce potential bias in self-reported data.

### 2.3 Measures

#### 2.3.1 Social demographic data

Social demographic data were collected in two dimensions: (1) demographic variables, such as age, gender, occupation, education level, monthly per capita household income, place of residence, marital status, religious beliefs, and medical insurance status; (2) disease-related variables, including clinical typology, duration of illness, age at onset, untreated period, number of hospitalizations, and medications used.

#### 2.3.2 Social Disability Screening Schedule

The SDSS, which was revised and validated by Xu et al. (13), assesses social functioning across 10 items, and each is rated on a 3-point scale (0 = no disability, 1 = some disability, and 2 = severe disability). A total score of ≥2 indicates severe social disability. The scale’s reliability was confirmed with a Kappa value that ranged between 0.6 and1.0.

#### 2.3.3 Brief Psychiatric Rating Scale

The BPRS, which was developed by Overall and Gorham, and revised by Zhang (14), assesses the severity of psychiatric symptoms across 18 items. Each item was rated on a 7-point scale (1 = asymptomatic and 7 = extremely severe). The scale was shown to have good reliability, with an inter-rater consistency of 0.787–0.970.

#### 2.3.4 The Medication Adherence Report Scale

The MARS-5, developed by Horne and Hankins (15), is a self-report measure designed to assess medication adherence using five items. Each item is rated on a 5-point Likert scale ranging from 1 (“Always”) to 5 (“Never”). The total score ranges from 5 to 25, with lower scores reflecting higher levels of adherence. The scale has demonstrated acceptable to good reliability, with Cronbach’s alpha coefficients ranging from 0.67 to 0.89 (16). The MARS is commonly employed in clinical and research settings due to its ease of use and robust psychometric properties. Permission to use the MARS-5 scale was obtained from the copyright holder. A copy of the permission letter is available upon request.

#### 2.3.5 Insight and Treatment Attitude Questionnaire

The ITAQ, which was developed by McEvoy et al. (17), assesses a patient’s insight into their illness, and attitude towards treatment. The 11-item questionnaire was rated on a 3-point scale (0 = no insight, 1 = partial insight, and 2 = complete insight). Higher scores indicated better insight. The scale’s reliability when measured by Cronbach’s alpha was 0.869 (18).

#### 2.3.6 Brief Assessment of Cognition in Schizophrenia

The BACS, which was developed by Sachs et al. (19), and adapted in China, evaluates cognitive function in schizophrenia patients across seven sub-tests (*e.g.* verbal memory, digit sequencing, and symbol coding). Higher scores reflected better cognitive functioning. The reliability of the assessment ranged between 0.65 and 0.92 (20).

#### 2.3.7 Social Support Rating Scale

The SSRS, which was developed by Xiao (21), assesses social support in three dimensions: objective support, subjective support, and utilization of support. The scale uses a 4-point Likert scale for most items, with higher scores indicating better perceived social support. The Cronbach’s alpha coefficient of the scale was 0.92.

#### 2.3.8 Perceived Devaluation-Discrimination Scale

The PDD, which was developed by Link in 2002, and translated into Chinese by Xu (22), measures perceived stigma in patients with mental illness. The scale includes 12 items rated on a 4-point Likert scale, with higher scores indicating greater stigma. A score of ≥25 indicated a high sense of stigma. The Cronbach’s alpha coefficient for the Chinese version was 0.76.

#### 2.3.9 Positive Psycap Questionnaire

The PPQ, which was developed by Zhang (23), evaluates psychological capital in four dimensions: self-efficacy, resilience, optimism, and hope. The 26-item questionnaire was rated on a 7-point Likert scale, with higher scores indicating better psychological health. The Cronbach’s alpha coefficient was 0.90.

#### 2.3.10 Positive and Negative Affect Scale

The PANAS, which was developed by Watson and Clark (24), assesses emotional states through 20 items divided into positive and negative affect categories. The scale uses a 5-point Likert scale, with higher scores indicating stronger emotional responses. The Cronbach’s alpha coefficient was 0.82.

### 2.4 Data analysis

Descriptive statistics, such as mean and standard deviation, were used for normally distributed data, while the median was reported for non-normally distributed data. Categorical variables were presented in frequency and composition ratio. Group comparisons for normally distributed data were conducted using ANOVA or *t*-test, while the Mann–Whitney *U*-test was used for non-normally distributed variables. Categorical variables were analyzed using chi-square test or Fisher’s exact test.

The required sample size was estimated using G*Power 3.1 software for binary logistic regression, with the objective of developing a risk prediction model for social disability in patients with schizophrenia. Based on a moderate expected effect size (odds ratio = 2.0), a two-sided significance level (α) of 0.05, and a statistical power of 80%, the minimum required sample size was calculated to be 134. The final sample size achieved in this study (n = 473) far exceeded this threshold, thereby ensuring sufficient statistical power to detect meaningful associations and enhancing the overall robustness and reliability of the model.

Variables that were statistically significant in the univariate analysis were included in the multivariate logistic regression model. Continuous variables were entered into the model as raw values, while categorical variables were converted into dummy variables. The variable selection criteria were set, as follows: α_in_ = 0.05 and α_out_ = 0.1. A risk prediction model for social disability in patients with schizophrenia was constructed based on independent risk factors and the corresponding regression coefficients. The formula is as follows:


$$\text{logit}(P)=In\frac{P}{1-P}$$

The predictive performance of the final model was assessed using the area under the receiver operating characteristic curve (AUC), with higher values indicating better discriminatory ability. The calibration of the model was evaluated using the Hosmer–Lemeshow goodness-of-fit test, which is a statistical method specifically designed to assess the alignment between predicted probabilities and observed outcomes in logistic regression models. A *p*-value of >0.05 indicates that there was no significant difference between the predicted and observed outcomes, suggesting that the model exhibited good calibration, and is reliable in estimating probabilities across a range of risk levels. SPSS version 22.0 was used for the univariate, multivariate, and logistic regression analyses, while R version 3.5.1 was employed to construct the nomogram prediction model.

## 3 Results

### 3.1 Demographic and clinical characteristics of the participants

A total of 500 questionnaires were distributed based on the predetermined inclusion and exclusion criteria. Among these questionnaires, 473 questionnaires were considered valid, yielding a valid recall rate of 94.60%. Among the participants, 56.00% were male, the average age was 29.31 ± 8.70 years old, 30.00% were unemployed, 33.40% had an education level of junior high school or below, and 17.50% had a monthly household per capita income of<1,000 yuan. The demographic and clinical information are presented in **Supplementary Table S1**.

### 3.2 Social disability situation of patients with schizophrenia

The study assessed social disability for the 473 patients diagnosed with schizophrenia. The median social disability score was 7 (range: 1–10), with scores spanning from 0 to 16. Social disability was measured using the SDSS, which consisted of 10 items rated on a 3-point scale (0–2): 0 indicates no disability, 1 indicates partial disability, and 2 indicates severe disability. Based on the SDSS scoring criteria, participants with a total score of ≥2 were categorized as having social disability. Consequently, the cohort was divided into two groups: social disability group (*n*=314) and non-social disability group (*n*=159). This resulted in a social disability rate of 66.40%. The detailed information on social disability scores is presented in **Supplementary Table S2**.

### 3.3 Univariate analysis of social disability in schizophrenia patients

The study conducted a univariate analysis to explore the factors associated with social disability in schizophrenia patients. The sociodemographic, illness-related, and psychosocial factors results are summarized below.

Sociodemographic factors: Statistically significant differences were identified between the social disability and non-social disability groups, in terms of occupation, level of education, monthly per capita household income, marital status, and place of residence (*p*<0.05). Patients in the social disability group tended to have lower educational attainment and lower income, and were more likely to be unmarried and reside in rural areas. The detailed findings are presented in **Table 1**.

**Table 1:** Univariate analysis of socio-demographic factors for the 473 schizophrenia patients (*n*, %).

Item	Disability group	Non-disability group	*x* ^2^-value	*p-*value
Gender				
Male	172 (36.40)	93 (19.60)	0.59	0.493
Female	142 (30.00)	66 (14.00)		
Age (year)			1.99	0.369
18~35	255 (53.90)	125 (26.40)		
36~45	42 (8.90)	20 (4.20)		
>45	17 (3.60)	14 (3.00)		
Ethnicity				
Han Chinese	308 (65.10)	154 (32.60)	0.71	0.519
Minor ethnic groups	6 (1.30)	5 (1.00)		
Occupation				
Student	37 (7.80)	18 (3.80)	108.88	<0.001
Worker/Farmer	58 (12.30)	35 (7.40)		
Public servants/Enterprises and institutions	12 (2.50)	37 (7.80)		
Unemployed	137 (29.00)	5 (1.00)		
Others	70 (14.90)	64 (13.50)		
Education level				
Junior high school and below	133 (28.10)	25 (5.30)	55.65	<0.001
Senior high school/Secondary vocational school	136 (28.80)	68 (14.40)		
College of technology and above	45 (9.50)	66 (13.90)		
Monthly per capita household income			83.97	<0.001
<1,000 yuan	79 (16.70)	4 (0.80)		
1,000–2,999 yuan	138 (29.20)	42 (8.90)		
3,000–4,999 yuan	82 (17.30)	80 (16.90)		
≥5,000 yuan	15 (3.20)	33 (7.00)		
Marital status			6.88	0.009
Married	234 (49.50)	100 (21.10)		
Others	80 (16.90)	59 (12.50)		
Place of residence			10.94	0.001
Urban	145 (30.70)	99 (20.90)		
Rural	169 (35.70)	60 (12.70)		
Religious belief			0.60	0.440
Yes	26 (5.50)	10 (2.10)		
No	288 (60.90)	149 (31.50)		
Medical insurance				
Yes	280 (59.20)	146 (30.90)	0.83	0.362
No	34 (7.20)	13 (2.70)		

Illness-related factors: The number of hospitalizations, age at onset, duration of illness, and brief mental symptom scores significantly differed between the two groups (p<0.05). The social disability group had more frequent hospitalizations, earlier age of onset, and longer illness durations. In addition, patients with poor medication compliance and lower insight scores had a higher risk of social disability. The detailed illness-related factors are presented in **Table 2**.

**Table 2:** Univariate analysis of factors related to illness for the 473 schizophrenia patients (*n*, %).

Items	Disability group	Non-disability group	*x* ^2^/*t*-value	*p-*value
Clinical typology			2.50	0.286
Paranoid	159 (33.60)	69 (14.60)		
Undefined	88 (18.60)	48 (10.10)		
Undifferentiated	67 (14.20)	42 (8.90)		
Times of hospitalization in the past year			2.79	0.095
≤1 time	287 (60.70)	152 (32.10)		
Two times and more	27 (5.70)	7 (1.50)		
Total times of hospitalization			27.70	*<*0.001
≤1 time	90 (19.00)	84 (17.80)		
2–4 times	184 (38.90)	66 (13.90)		
≥5 times	40 (8.50)	9 (1.90)		
Age of onset			19.12	*<*0.001
≤18 years old	92 (19.50)	18 (3.80)		
>18 years old	222 (46.90)	141 (29.80)		
Duration of illness (year)			41.18	*<*0.001
0–3	164 (34.70)	127 (26.80)		
4–10	110 (23.30)	32 (6.70)		
>10	40 (8.50)	0 (0.00)		
Untreated period (months)			37.48	*<*0.001
0	190 (40.30)	139 (29.40)		
1–12	116 (24.50)	18 (3.80)		
13–24	4 (0.80)	2 (0.40)		
≥25	4 (0.80)	0 (0.00)		
Medication status			0.26	0.625
Single medication	29 (6.10)	17 (3.60)		
Combined medication	285 (60.30)	142 (30.00)		
Brief Mental Symptom Score	44.61 ± 9.29	25.14 ± 4.52	−24.96	*<*0.001
Medication adherence			72.67	*<*0.001
<20	309 (65.3)	117(24.70)		
≥20	5 (1.10)	42 (8.90)		
Insight and Treatment Attitude Score			16.26	*<*0.001
≤5	17 (3.60)	1 (0.20)		
6–19	297 (62.80)	153 (32.30)		
≥20	0 (0.00)	5 (1.10)		
BACS total score	195.82 ± 30.97	269.42 ± 22.02	26.74	*<*0.001

BACS, Brief Assessment of Cognition in Schizophrenia.

Psychosocial factors: Statistically significant differences were identified in social support (SSRS score), stigma (PDD score), psychological capital, and emotional state between the two groups (*p*<0.05). Patients in the social disability group exhibited lower social support and psychological capital scores, and reported higher levels of perceived stigma and negative emotions. Furthermore, the positive and negative emotion scores were significantly different between the groups. The detailed psychosocial factors are presented in **Table 3**.

**Table 3:** Univariate analysis of psychosocial factors for the 473 schizophrenia patients (*n*, %).

Items	Disability group	Non-disability group	*x* ^2^/*t*-value	*p-*value
SSRS score	30.01 ± 4.18	38.18 ± 3.52	21.12	*<*0.001
Sense of Stigma Score			352.87	*<*0.001
<25	23 (4.90)	152 (32.10)		
≥25	291 (61.50)	7 (1.50)		
Psychological Capital Score			361.68	*<*0.001
≤111	291 (61.50)	5 (1.10)		
112–132	21 (4.40)	130 (27.50)		
≥133	2 (0.40)	24 (5.10)		
Positive Emotion Score	24.05 ± 3.30	33.28 ± 4.71	24.74	*<*0.001
Negative Emotion Score	25.61 ± 3.88	17.74 ± 2.99	−22.44	*<*0.001

SSRS, Social Support Rating Scale.

### 3.4 Multivariate analysis of social disability in schizophrenia patients

Variables that were statistically significant in the univariate analysis (occupation, level of education, monthly per capita household income, marital status, place of residence, total hospitalizations, age of onset, duration of illness, untreated period, brief mental symptom score, medication compliance, insight, cognitive function [BACS score], social support [SSRS score], stigma [PDD score], psychological capital, and emotional state) were included in the logistic regression analysis (the value assignment is presented in **Supplementary Table S3**). The results indicated that the following six factors were the independent predictors of social disability in schizophrenia patients: brief mental symptom score, medication compliance, BACS score, stigma score, SSRS score, and psychological capital score. These factors were identified to have statistically significant regression coefficients, suggesting that these are critical predictors of social dysfunction. The detailed regression results are presented in **Table 4**.

**Table 4:** Multivariate analysis of risk factors for social disability for the 473 schizophrenia patients.

Regression variable	Regression coefficient (B)	Wald Chi-square	OR value	95% CI	*p-*value
Brief Mental Score	0.278	8.159	1.321	1.130–1.690	0.004
Medication adherence (MARS-5)	−3.337	4.582	0.036	0.001–0.492	0.032
Cognitive Function Total Score (BACS)	−0.064	5.201	0.938	0.874–0.982	0.023
Social Support Total Score (SSRS)	−0.304	7.665	0.738	0.569–0.905	0.006
Sense of Stigma Score	4.519	8.117	1.759	3.013–6.457	0.004
Psychological Capital Score	−3.415	7.826	0.039	0.002–0.284	0.005
Constant	−3.338	5.190			0.023

OR, odds ratio; 95% CI, 95% confidence interval.

### 3.5 Development and visual representation of the risk prediction model

A risk prediction model for social disability in schizophrenia patients was developed based on the logistic regression analysis. The model included six independent risk factors (brief mental symptom score, medication compliance, cognitive function [BACS score], stigma [PDD score], social support [SSRS score], and psychological capital score), along with the corresponding regression coefficients. The prediction formula was, as follows: Logit (r disability) = −3.338 + 0.278 × brief mental symptom score + 4.519 × stigma score − 3.33 × MARS-5 score − 0.064 × BACS score − 0.304 × SSRS score − 3.415 × psychological capital score.

**Figure 1:**
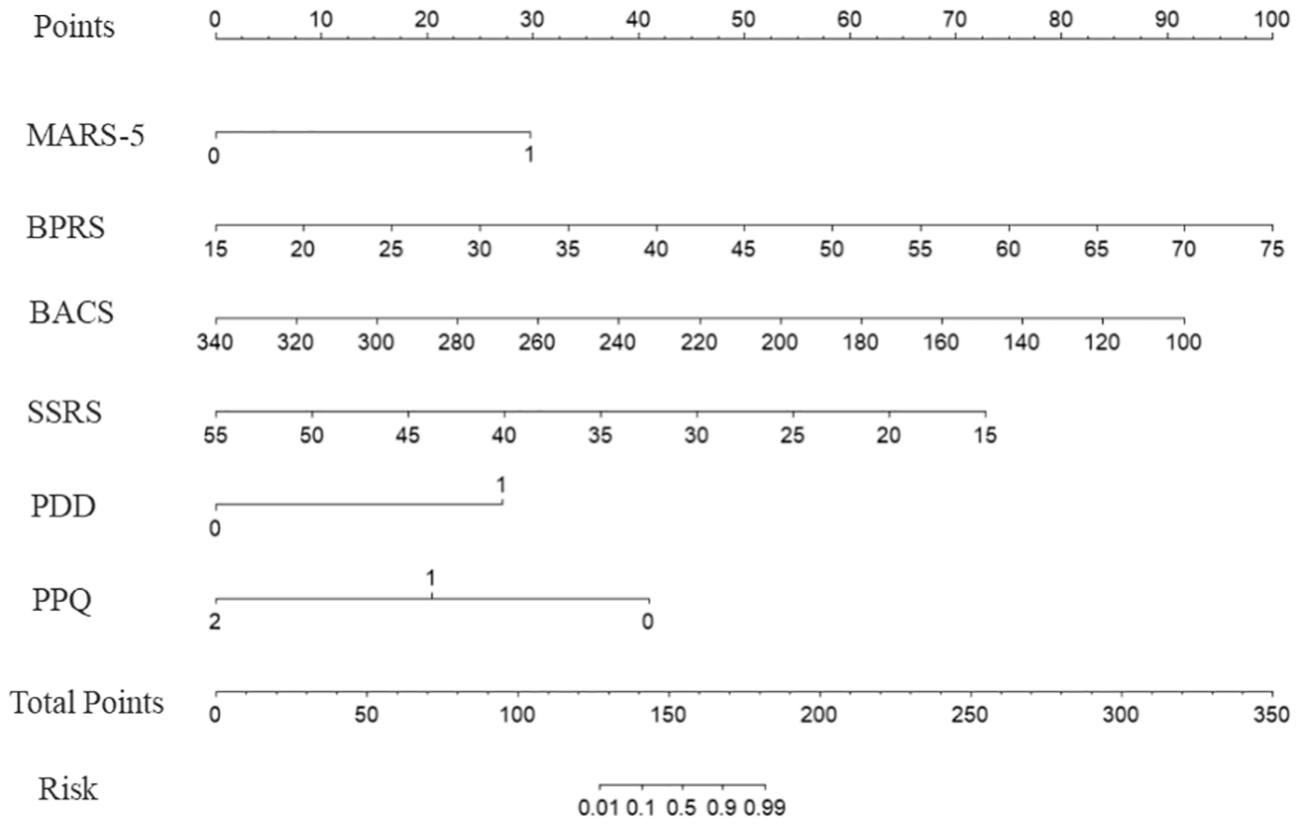
Nomogram for predicting the risk of social disability in patients with schizophrenia. The model incorporates six predictors: medication adherence (MARS-5, dichotomized as<20 = 0, ≥20 = 1), symptom severity (Brief Psychiatric Rating Scale, BPRS), cognitive function (Brief Assessment of Cognition in Schizophrenia, BACS), social support (Social Support Rating Scale, SSRS), perceived stigma (Perceived Devaluation-Discrimination, PDD, dichotomized as<25 = 0, ≥25 = 1), and psychological resilience (Psychological Capital Questionnaire, PPQ, categorized as ≤111 = 0, 112–132 = 1, ≥133 = 2). Each predictor is assigned a corresponding score, which is summed to obtain the total points. The total points are then mapped onto the risk probability scale at the bottom to estimate the likelihood of social disability. Higher total points indicate an increased risk of social disability.

These scores were assigned to each factor based on the model, and an alignment diagram was created to visually represent the risk prediction model for schizophrenia-related social disability. The specific assignments for each variable are presented in **Supplementary Table S4**, and the alignment diagram is presented in **Figure 1**.

### 3.6 Performance evaluation of the risk prediction model

**Figure 2:**
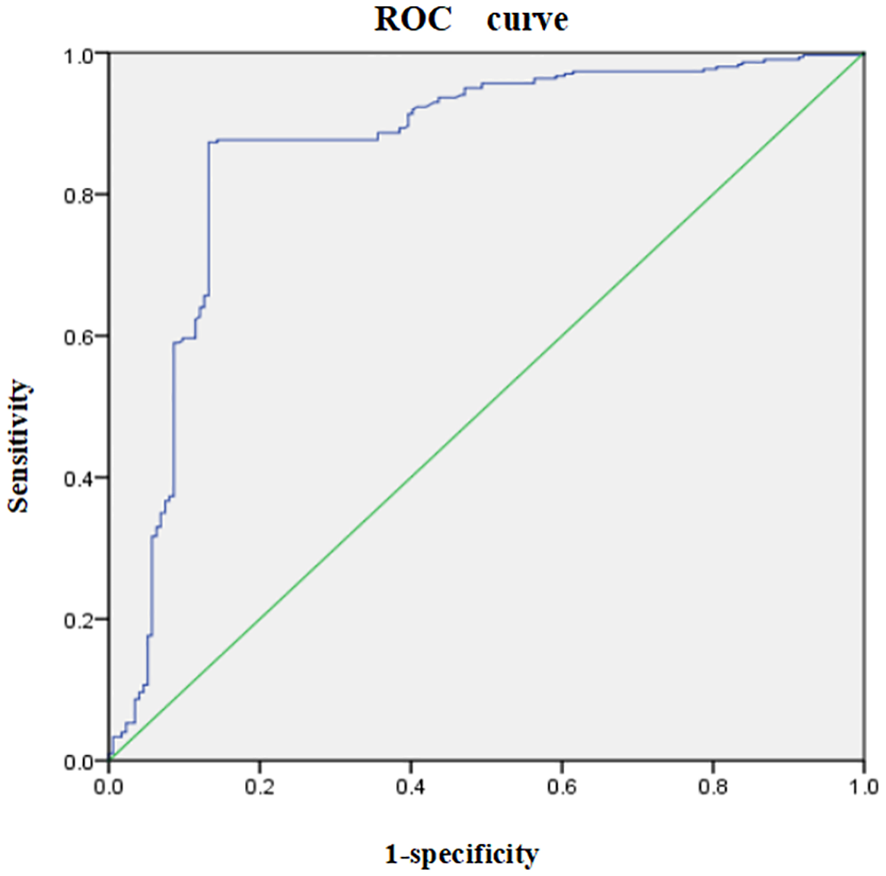
Receiver operating characteristic (ROC) curve for the risk prediction model for social disability in schizophrenia patients. The ROC curve illustrates the trade-off between sensitivity (y-axis) and 1-specificity (x-axis) across different threshold values. The area under the curve (AUC) represents the model’s ability to discriminate between patients with and without social disability, with an AUC close to 1 indicating excellent predictive performance.

Discriminatory power: The discriminatory power of the model was evaluated using the AUC. The results indicated an AUC of 0.860 (95% CI: 0.820–0.899, *p*<0.001), suggesting that the model has strong discriminatory ability. The optimal cut-off value was −30.13, with a Youden index of 0.741. The sensitivity and specificity was 0.873 and 0.868, respectively. The receiver operating characteristic (ROC) curve is presented in **Figure 2**.

Calibration capabilities: The sensitivity and specificity of the model were 0.873 and 0.868, respectively. The calibration was deemed good, as indicated by the Hosmer–Lemeshow test (*x^2^ =* 5.746, *p*=0.783), indicating that there was no statistically significant difference between the predicted and actual probabilities of social disability occurrence. This suggests that the calibration capabilities of the model are reliable.

## 4 Discussion

### 4.1 Summary of key findings

The present study developed and validated a risk prediction model for social disability in schizophrenia patients, incorporating six key independent risk factors: psychiatric symptoms, medication compliance, cognitive function, sense of stigma, social support, and psychological capital. The model demonstrated strong predictive accuracy, with an AUC of 0.860, a sensitivity of 0.873, and a specificity of 0.868. These findings suggest that the model can effectively identify schizophrenia patients at high risk for social disability, providing a foundation for early and targeted interventions aimed in improving patient outcomes. Although disease-related factors—such as disease severity, age at onset, illness duration, duration of untreated psychosis, and number of hospitalizations—were not identified as independent predictors of social functioning impairment in this study, our findings are consistent with previous research. For instance, regarding disease severity, our results align with those reported by Saris et al., who found no significant association between disease severity and overall social functioning scores. Instead, emotional variables—such as higher levels of positive affect and lower levels of depression and anxiety—were more strongly correlated with better social functioning (25). Similarly, a multicenter cohort study conducted across six European centers reported that neither age at onset nor the duration of untreated psychosis was predictive of long-term social functioning deficits (26). With respect to illness duration, this may be explained by the conceptualization of social dysfunction as a relatively independent domain within the course of schizophrenia. Illness duration tends to be more closely associated with intrinsic biological factors, whereas impairments in social functioning are often shaped by an individual’s acquired level of personal and social competence at the time of disease onset (27). In particular, the quality and extent of premorbid social relationships—especially meaningful connections outside of the family context—have been shown to be significant prognostic indicators of favorable long-term clinical and social outcomes in individuals with schizophrenia (28).

### 4.2 Interpretation of key independent risk factors

#### 4.2.1 Psychiatric symptoms

The severity of psychiatric symptoms was identified as a significant predictor of social disability among patients with schizophrenia. Psychiatric symptoms refer to the observable behavioral manifestations of underlying psychological disturbances and are typically categorized into positive symptoms, negative symptoms, and cognitive impairments (29). Among these, negative symptoms and cognitive deficits have been consistently recognized as key contributors to impaired social functioning (30, 31). Our findings further support this association, demonstrating a clear positive correlation between overall symptom severity and the degree of social dysfunction, which was incorporated into the risk prediction model for social disability. In particular, negative symptoms—such as motivational deficits, social withdrawal, and blunted affect—directly compromise an individual’s capacity to initiate and maintain interpersonal relationships, thereby resulting in pronounced social impairment (32–34). Furthermore, these symptoms reduce social skills and interest in social activities, resulting in lower engagement and enjoyment during interpersonal interactions (35). Moreover, negative symptoms are associated to diminished social cognition, which exacerbate challenges in understanding and responding to social cues, thereby deepening social functional impairments (36). Addressing these symptoms through targeted interventions, including behavioral therapies, would play a crucial role in reducing social disability, and enhancing the quality of life of schizophrenia patients.

#### 4.2.2 Medication adherence

Poor medication adherence is another significant predictor of social disability in schizophrenia. In this study, approximately 45.80% of patients exhibited poor adherence to their medication regimens, resulting in symptom exacerbation, increased relapse rates, and higher hospitalization frequencies—all of which contribute to social dysfunction (37, 38). A robust relationship exists between medication adherence and social functioning, as poor compliance can lead to a cycle of worsening symptoms and escalating social disability. Interventions aimed at improving medication adherence—through health education, cognitive interventions, and peer support—could reduce these risks, promoting sustained social recovery. Enhancing medication compliance is not only vital for symptom control but also plays a key role in preventing relapses and minimizing the risk of social disability.

#### 4.2.3 Cognitive function

Cognitive impairments, which are prevalent in schizophrenia, were identified as critical factors contributing to social disability. These impairments, which include deficits in attention, memory, executive functions, and social cognition, significantly hinder the patients’ ability to interpret and respond to social cues, perform daily tasks, and maintain social and occupational roles (39). Cognitive deficits mediate the relationship between neurocognitive abilities and functional outcomes, with social cognitive deficits, such as emotion recognition and theory of mind, playing a particularly crucial role (40). These cognitive challenges persist across all phases of illness, including remission, and are strongly correlated with higher levels of socio-occupational dysfunction and social disability (41). Studies have shown that cognitive rehabilitation interventions, such as cognitive remediation therapy, can enhance cognitive function and improve social outcomes in schizophrenia patients (42, 43). Cognitive rehabilitation may therefore be a key intervention to reduce social disability by improving cognitive and social functioning.

#### 4.2.4 Sense of stigma and psychosocial factors

The sense of stigma experienced by 63% of patients was strongly associated to social disability. The sense of stigma in individuals with schizophrenia significantly contributed to social disability by fostering internalized negative perceptions, reducing self-esteem, and impairing quality of life. Furthermore, patients who experienced stigma were more likely to withdraw socially, suffer from depression, and exhibit avoidance behaviors, exacerbating their symptoms, and creating a cycle of worsening disability (44). Moreover, this internalized stigma correlated with lower social integration, heightened feelings of alienation, and social anxiety, further hindering rehabilitation efforts and personal fulfillment (45, 46). Addressing stigma through education and social support is crucial to reducing its negative impact on social functioning. In addition, enhancing social support systems, both from families and communities, can foster better social reintegration, and reduce the likelihood of social disability (47, 48).

#### 4.2.5 Psychological capital

Psychological capital, including self-efficacy, resilience, optimism and hope, was identified as a protective factor. Patients with higher psychological capital were more likely to exhibit better social functioning (49). Deficient psychological capital can directly contribute to social disability in schizophrenia by limiting an individual’s capacity to cope with the challenges of social interactions and functional roles. Deficits in psychological traits, such as resilience, self-efficacy and hope, impair a patients ability to recover from social setbacks, actively participate in social environments, and manage the demands of everyday life. Lack of self-efficacy often leads to avoidance behaviors and withdrawal from social opportunities, which in turn exacerbates isolation, and reduces functional independence (50). Similarly, low resilience hinder individuals from overcoming failures or negative experiences in social contexts, reinforcing cycles of disengagement and social dysfunction. Interventions aimed at fostering positive psychological traits through therapies, such as group therapy or positive thinking training, can improve the ability of patients to cope with stress, and engage in social interactions, promoting social recovery, and reducing the likelihood of social disability (23).

### 4.3 Clinical application and significance of the risk prediction models

The risk prediction model developed in this study holds considerable clinical value by enabling the early identification of schizophrenia patients at high risk for social disability. In clinical settings, the model can be integrated into electronic medical record systems to automatically flag high-risk individuals during routine psychiatric assessments. For these patients, clinicians may prioritize tailored interventions. Moreover, the model supports healthcare providers in implementing personalized strategies—such as cognitive rehabilitation, medication adherence programs, and psychosocial support—targeted to address specific risk factors contributing to social dysfunction, thereby mitigating its progression. By quantifying individual risk levels, the model facilitates more precise clinical decision-making and supports the development of personalized treatment plans. Its potential integration into routine clinical workflows offers a structured approach to patient management. By identifying individuals most likely to experience social dysfunction, healthcare resources can be allocated more efficiently, ensuring timely delivery of specialized services to those in greatest need.

### 4.4 Limitations and future directions

Despite the strengths of the risk prediction model, there were several limitations that should be addressed. First, the study did not incorporate biochemical markers or neuroimaging data, which could have improved the model’s predictive accuracy. Second, as a single-center study, the model was not validated with external datasets, which limits its generalizability to broader populations. Third, the cross-sectional design of the study introduced inherent biases, including reliance on existing records, and the possibility of missing data. Fourth, the study only investigated the medication types and discontinuation times, without considering the potential effects of dosage, which may have influenced the results. In order to address these limitations, future research should prioritize validating the model using external datasets from diverse populations, in order to enhance its robustness and applicability. This study did not assess the role of psychotherapy and counseling as protective factors, which are commonly used without prescription to manage symptoms. As such, relevant data were not collected. Future research should consider including these interventions to better understand their potential impact on symptom alleviation.In addition, multi-center, prospective studies and randomized controlled trials are essential to further evaluate the model’s effectiveness, and investigate causal relationships. Incorporating genetic and neurobiological factors would also refine the model’s predictive power. Finally, accounting for medication dosage effects in future studies could provide a more comprehensive understanding of its impact on social disability risk.

## 5 Conclusions

The present study developed a risk prediction model for social disability in schizophrenia patients, and identified six key independent risk factors that influence social functioning. The model shows potential for clinical application, particularly in early risk screening and personalized intervention. Targeted interventions that address these risk factors may contribute to improved social outcomes for schizophrenia patients. However, additional research is required to confirm its long-term effectiveness in enhancing patient quality of life.

## Data Availability

The raw data supporting the conclusions of this article will be made available by the authors, without undue reservation.
