# Supplementary material for: Development and evaluation of a risk prediction model for social disability in schizophrenia patients
Source: Front Psychiatry. 2025 Oct 7;16:1588849. doi: 10.3389/fpsyt.2025.1588849 (PMC12538653; doi:10.3389/fpsyt.2025.1588849)
Supplement: Supplementary file 1 [file Table1.doc]

**Table S1. General information of the 473 schizophrenia patients ($\overline{x}$±*s,* [*n*, %]).**

| **Item** | **Number of cases** |
| --- | --- |
| Gender |  |
| Male | 265 (56.00) |
| Female | 208 (44.00) |
| Age (years) | 29.31 ± 8.70 |
| Ethnicity |  |
| Han Chinese | 462 (97.70) |
| Minor ethnic groups | 11 (2.30) |
| Occupation |  |
| Student | 55 (11.60) |
| Worker/Farmer | 93 (19.70) |
| Public servants/Enterprises and institutions | 49 (10.40) |
| Unemployed | 142 (30.00) |
| Others | 134 (28.30) |
| Education Level |  |
| Junior high school and below | 158 (33.40) |
| Senior high school/Secondary vocational school | 204 (43.10) |
| College of technology and above | 111 (23.50) |
| Monthly per capita household income |  |
| <1,000 yuan | 83 (17.50) |
| 1,000-2,999 yuan | 180 (38.10) |
| 3,000-4,999 yuan | 162 (34.20) |
| ≥5,000 yuan | 48 (10.10) |
| Marital status |  |
| Married | 139 (29.40) |
| Others | 334 (70.60) |
| Place of residence |  |
| Urban | 244 (51.60) |
| Rural | 229 (48.40) |
| Religious belief |  |
| Yes | 36 (7.60) |
| No | 437 (92.40) |
| Medical insurance |  |
| Yes | 426 (90.10) |
| No | 47 (9.90) |

**Table S2. Assessment of social disability for the 473 schizophrenia patients (*n*, %)**

| **Item** | **No disability (0 point)** | **Partial disability (1 point)** | **Serious disability (2 points)** |
| --- | --- | --- | --- |
| 1. Occupation and work | 63 (13.30) | 172 (36.40) | 96 (20.30) |
| 2. Marital function | 116 (24.50) | 53 (11.20) | 11 (2.30) |
| 3. Parental function | 67 (14.20) | 42 (8.90) | 17 (3.60) |
| 4. Social withdrawal | 160 (33.80) | 153 (32.30) | 160 (33.80) |
| 5. Extra-family social activity | 162 (34.20) | 164 (34.70) | 147 (31.10) |
| 6. Too little activity within family | 215 (45.40) | 174 (36.80) | 84 (17.80) |
| 7. Familial function | 239 (50.50) | 172 (36.40) | 62 (13.10) |
| 8. Self-care for personal life | 384 (81.20) | 84 (17.80) | 5 (1.00) |
| 9. Interest and concern on the external world | 186 (39.30) | 144 (30.40) | 143 (30.30) |
| 10. Responsibility and planning | 161 (34.00) | 174 (36.80) | 138 (29.20) |

**Notes:** (1) 142 patients were unemployed; (2) 293 patients were unmarried; (3) 347 patients were without children

**Table S3. Multivariate logistic regression value assignment for risk factors for social disability in schizophrenia patients.**

| **Factor** | **Variable name** | **Assignment description** |
| --- | --- | --- |
| Result | Y | 0 = social disability, 1 = non-social disability |
| Occupation | X_1_ | 0 = Enterprises/Institutions, 1 = student, 2 = Worker/Farmer, 3 = Others, 4 = Unemployed |
| Education level | X_2_ | 0 = College of technology and above, 1 = Senior high school/Secondary vocational school, 2 = Junior high school and below |
| Monthly per capita household income (yuan) | X_3_ | 0 = ≥5,000, 1 = 3,000-4,999, 2 = 1,000-2,999, 1 = <1,000 |
| Marital status | X_4_ | 0 = Married, 1 = others |
| Place of residence | X_5_ | 0 = urban, 1 = rural |
| Total times of hospitalization | X_6_ | 0 = ≤1 time, 1 = 2-4 times, 2 = ≥5 times |
| Age of onset | X_7_ | 0 = >18 years old, 1 = ≤18 years old |
| Duration of illness (year) | X_8_ | 0 = 0-3, 1 = 4-10, 2 = >10 |
| Untreated period (month) | X_9_ | 0 = 0, 1 = 1-12, 2 = 13-24, 3 = ≥25 |
| Brief Mental Symptom Score | X_10_ | input value variable |
| Medication compliance | X_11_ | 0 = 8, 1 = 6-7, 2 = <6 |
| Insight and Treatment Attitude Score | X_12_ | 0 = ≥20, 1 = 6-19, 2 = ≤5 |
| BACS total score | X_13_ | input value variable |
| SSRS total score | X_14_ | input value variable |
| Devaluation-Discrimination Perception Score | X_15_ | 0 = <25, 1 = ≥25 |
| Psychological Capital Score | X_16_ | 0 = ≥133, 1 = 112-132, 2 = ≤111 |
| Positive Emotion Score | X_17_ | input value variable |
| Negative Emotion Score | X_18_ | input value variable |

**Table S4. Process of assigning scores to different factors and results.**

| **Factor** | **Regression coefficient (B)** | **Wald Chi-square** | **OR value** | **95% confidence interval** | **Score assignment** |
| --- | --- | --- | --- | --- | --- |
| Brief Mental Symptom Score | 0.278 | 7.866 | 1.320 | 1.087-1.604 |  |
| Medication compliance |  |  |  |  |  |
| <6 | - | Reference | Reference | - | 0 |
| 6-7 | 7.985 | 2.528 | 37.581 | 6.562-55.873 | 63 |
| 8 | 5.032 | 1.175 | 13.267 | 2.173-13.636 | 100 |
| Cognitive Function Total Score | -0.065 | 5.142 | 0.937 | 0.886-0.991 |  |
| Social Support Total Score | -0.306 | 7.819 | 0.737 | 0.594-0.913 |  |
| Sense of Stigma Score |  |  |  |  |  |
| <25 | - | Reference | Reference | - | 0 |
| ≥25 | -4.643 | 7.524 | 2.451 | 1.872-5.266 | 58 |
| Psychological Capital Score |  |  |  |  |  |
| ≤111 | 0 | Reference | Reference | - | 0 |
| 112-132 | 6.694 | 1.081 | 7.403 | 4.539-24.492 | 83 |
| ≥133 | 3.348 | 0.273 | 5.441 | 2.324-5.692 | 42 |

**Notes:** OR, odds ratio
